# Melittin, a honeybee venom derived peptide for the treatment of chemotherapy-induced peripheral neuropathy

**DOI:** 10.1007/s12032-021-01496-9

**Published:** 2021-04-02

**Authors:** Tenzin Tender, Rakesh Ravishankar Rahangdale, Sridevi Balireddy, Madhavan Nampoothiri, K. Krishna Sharma, Hariharapura Raghu Chandrashekar

**Affiliations:** 1 Department of Pharmaceutical Biotechnology, Manipal College of Pharmaceutical Sciences, Manipal Academy of Higher Education, Manipal, 576104 Karnataka India; 2 Department of Pharmacology, Manipal College of Pharmaceutical Sciences, Manipal Academy of Higher Education, Manipal, 576104 Karnataka India; 3 Department of Ophthalmology and Biochemistry, University of Missouri - Columbia School of Medicine, Columbia, MO 65211 USA

**Keywords:** Chemotherapy, Peripheral neuropathy, Melittin, Haemolysis, α-Crystallin

## Abstract

**Abstract:**

Chemotherapy-induced peripheral neuropathy (CIPN) is the most prevalent neurological complication of cancer treatment which involves sensory and motor nerve dysfunction. Severe CIPN has been reported in around 5% of patients treated with single and up to 38% of patients treated with multiple chemotherapeutic agents. Present medications available for CIPN are the use of opioids, nonsteroidal anti-inflammatory agents, and tricyclic antidepressants, which are only marginally effective in treating neuropathic symptoms. In reality, symptom reappears after these drugs are discontinued. The pathogenesis of CIPN has not been sufficiently recognized and methods for the prevention and treatment of CIPN remain vulnerable to therapeutic problems. It has witnessed that the present medicines available for the disease offer only symptomatic relief for the short term and have severe adverse side effects. There is no standard treatment protocol for preventing, reducing, and treating CIPN. Therefore, there is a need to develop curative therapy that can be used to treat this complication. Melittin is the main pharmacological active constituent of honeybee venom and has therapeutic values including in chemotherapeutic-induced peripheral neuropathy. It has been shown that melittin and whole honey bee venom are effective in treating paclitaxel and oxaliplatin-induced peripheral neuropathy. The use of melittin against peripheral neuropathy caused by chemotherapy has been limited despite having strong therapeutic efficacy against the disease. Melittin mediated haemolysis is the key reason to restrict its use. In our study, it is found that α-Crystallin (an eye lens protein) is capable of inhibiting melittin-induced haemolysis which gives hope of using an appropriate combination of melittin and α-Crystallin in the treatment of CIPN. The review summarizes the efforts made by different research groups to address the concern with melittin in the treatment of chemotherapeutic-induced neuropathy. It also focuses on the possible approaches to overcome melittin-induced haemolysis.

**Graphic Abstract:**

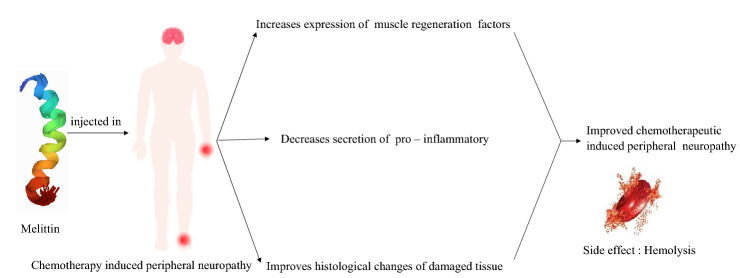

## Introduction

Cancer continues to be the leading cause of morbidity and mortality in the globe [1, 2] with mortality increases of about 25.0% since the 1990s and estimates as much as ≥ 23 million annually by 2030 [3, 4]. Most of the cancer patients are either treated with curative or palliative chemotherapy throughout the treatment. While chemotherapy has significantly enhanced survival rates in several cancer forms, chemotherapy-induced peripheral neuropathy (CIPN) is a common and serious clinical problem that affects many patients receiving cancer treatment.

Based on the particular chemotherapeutic compounds, there can be different types of neuropathies: large and small fiber, sensory and/or motor, demyelinating and axonal, cranial, and autonomic [5]. Chemotherapy’s effects on the nervous system vary across different types of medications in which drug’s physical and chemical properties, whether used in single or combined doses have a significant role [6]. The prevalence of CIPN is based on the person, with rates reported ranging from 19% to over 85% [7] and it is the largest for platinum-based medications (70–100%) followed by taxanes (11–87%), ixabepilone (60–65%), and thalidomide and its analogs (20–60%), (6). Toxicity can occur either with a large single dosage or after combined exposure. The signs observed differ in severity, duration, and range from intense temporary warm stimuli to persistent peripheral nerve changes caused by severe pain and lasting nerve damage. Recent reports placed the prevalence of CIPN at about 68.1% when assessed in the first month following chemotherapy, 60% at third months, and 30% after sixth months [8]. Chemotherapeutics, such as oxaliplatin and paclitaxel, are major sources of neuropathic pain caused by the medication. CIPN results in impairment in the quality of life of cancer survivors patients and often lead to change in either the drug dosages or combinations especially in patients with acute neuropathy. CIPN is seen as an acceptable, unavoidable complication of chemotherapy by caregivers, and is also necessary to save the life of patients [9]. In contrast, CIPN was seen by cancer patients as an often difficult chemotherapeutic complication affecting their quality of life [10]. CIPN put up to delay functional recovery, decrease treatment tolerability causing symptom distress in cancer patient [9]. Clinical symptoms of CIPN mainly involve sensory axonal neuropathy with occasionally motor and autonomic indulgence affecting predominantly the arm and leg of the patients. Usually, sensory fibers are mostly affected but sometimes cause a sensorimotor pattern by cytostatic agents. Typical symptoms include numbness, paraesthesia, lancinating pain, abnormal gait, and motor weakness. It is important to keep in mind that CIPN will extend beyond antineoplastic therapy for several years and is associated with an elevated risk of falls [11]. Hence, cancer-related neuropathy is considered a major adverse outcome for cancer patients. No drug can currently be proposed as a gold standard to either prevent CIPN or treat its symptoms, and the only preventive strategy remains to modify the chemotherapeutic drug dose. According to the American Society of Clinical Oncology (ASCO), clinicians may offer duloxetine along with tricyclic antidepressants, gabapentin, or pregabalin to manage CIPN-induced symptoms. However, these agents do not affect motor symptoms or negative sensory symptoms [12, 13]. Therefore, a curative medication needs to be established that can be used to treat this complication. The review summarises the numerous research groups' attempts to subdue the chemotherapy-induced neuropathy using melittin and reflects on the potential strategies of resolving haemolysis caused by melittin.

## Pathophysiology of chemotherapy-induced neuropathy

CIPN can induce severe pain and impairment leading to significant loss of functional ability and reduced quality of life. Neurotoxic chemotherapy can cause peripheral nerve structural damage resulting in aberrant somatosensory processing of the peripheral and central nervous system [14]. It is rare to reach the firing threshold in normal primary afferent neurons without the input of a stimulus. However, after nerve injury it is believed that most damaged axons and related cell bodies in the diagnosis-related groups experience an increase in their intrinsic electrical excitability. As a consequence, they start generating impulse discharge spontaneously or with only minimal stimulation associated with the site of injury resulting in excessive spontaneous and stimulus-related electrical impulses feeding into the central nervous system [15]. There are four main substance groups namely platinum-based antineoplastic agents, taxanes, epothilones and immunomodulatory drugs that cause damage to peripheral sensory, motor and autonomic neurons, which result in the development of CIPN. Following are the mechanisms of CIPN induced by these drugs.

### Platinum-based antineoplastics (oxaliplatin, cisplatin, and carboplatin)

The exact cause of chemotherapeutics-induced peripheral neuropathy by platinum-based agents is not yet well understood; however, their antitumor pathways tend to be responsible for neurotoxic activity because chemotherapy induces different modifications in the configuration or functions of neuronal and glial cells [16]. A number of changes in intracellular organelles (particularly mitochondria), membrane receptors, and ion channels are caused by chemotherapeutic agents, followed by changes in intracellular homeostasis, signaling, and neurotransmission, all of which may lead to neuroinflammation, damage to DNA, and axonal degeneration.

**Fig. 1:**
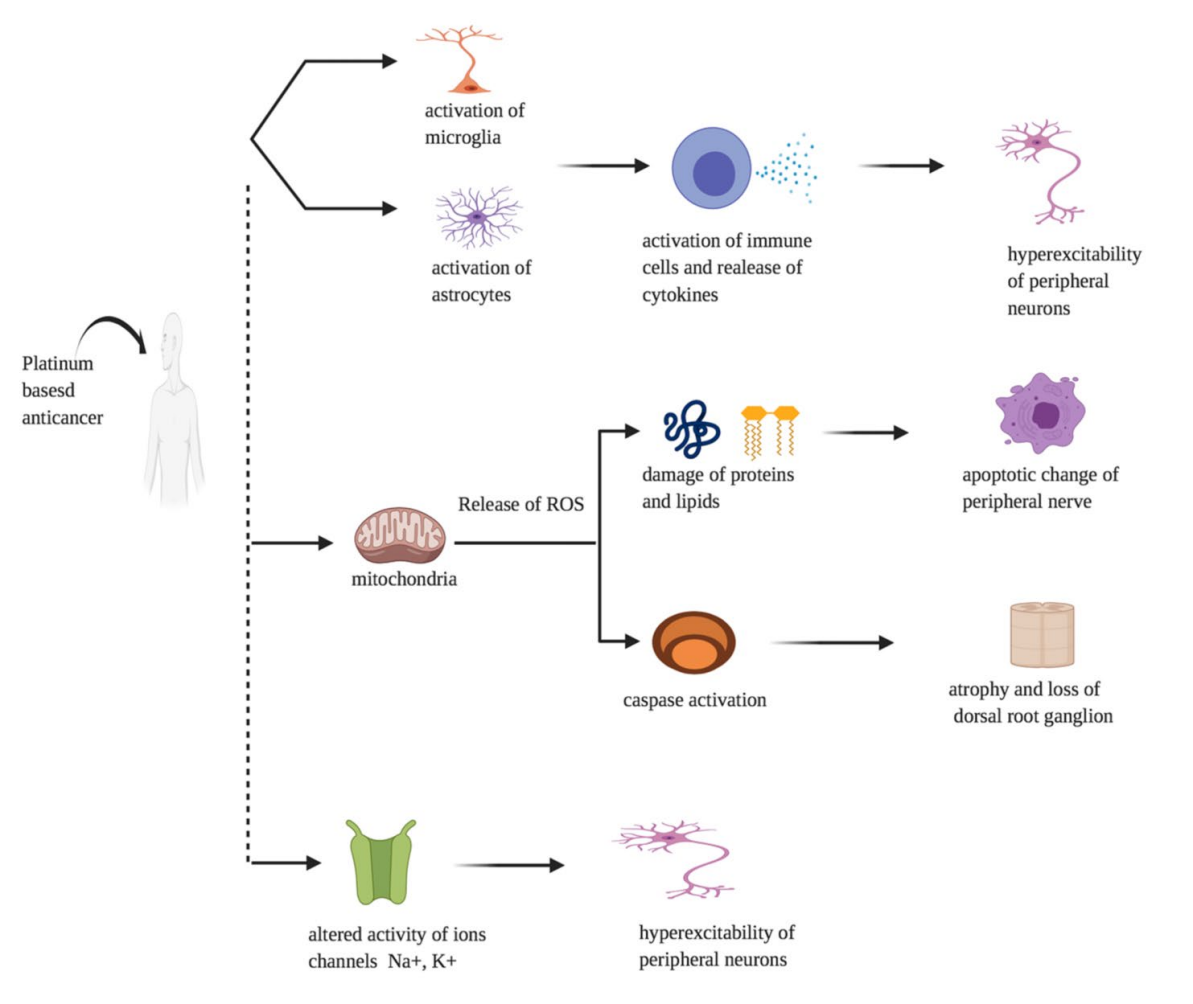
Pathophysiology of platinum-based antineoplastics-induced peripheral neuropathy. Effect of platinum-based anticancer on microglia, astrocytes, mitochondria and ions channels leading to peripheral neuropathy

Platinum-based drugs stimulates glial cells contributing to the activation of immune cells and the release and elevation of pro-inflammatory cytokines (interleukins and chemokines), resulting in nociceptor sensitization and peripheral neuron hyperexcitability, and disrupting the blood–brain barrier (together with ROS). Mitochondrial disruption induced by platinum-based medications contributes to enhanced development of reactive oxygen species (ROS), resulting in neuronal harm to enzymes, proteins, and lipids, as well as calcium homeostasis dysregulation, which causes apoptotic adjustments in peripheral nerves and dorsal root ganglion (DRG) cells. Platinum-based drugs also change the ion channels of Na+, K+, and TRP activity, resulting in peripheral neurons becoming hyperexcitable (Fig. 1). Many of the mechanisms mentioned above have the capacity to change peripheral neuron excitability [16, 17].

### Taxanes (paclitaxel, docetaxel and cabazitaxel) and epothilones (ixabepilone)

Taxanes constitute acts on microtubules and interfere with the normal cycle of microtubule depolymerization and repolymerization leading to the death of cancer cells. Taxanes-induced peripheral neuropathy by disrupting microtubule which impairs axonal transport and contribute to Wallerian degeneration, altered ion channel function, and peripheral neuron hyperexcitability. Taxanes also alter the expression and function of the ion channels (Na^+^, K^+^, and TRP) resulting in the hyperexcitability of peripheral neurons. Taxane-induced mitochondrial damage contributes to the increased production of reactive oxygen species (ROS), which leads to enzyme, protein and lipid damage as well as the dysregulation of calcium homeostasis within neurons resulting in apoptotic changes and the demyelination of peripheral nerves. These processes alter the excitability of peripheral neurons.

**Fig. 2:**
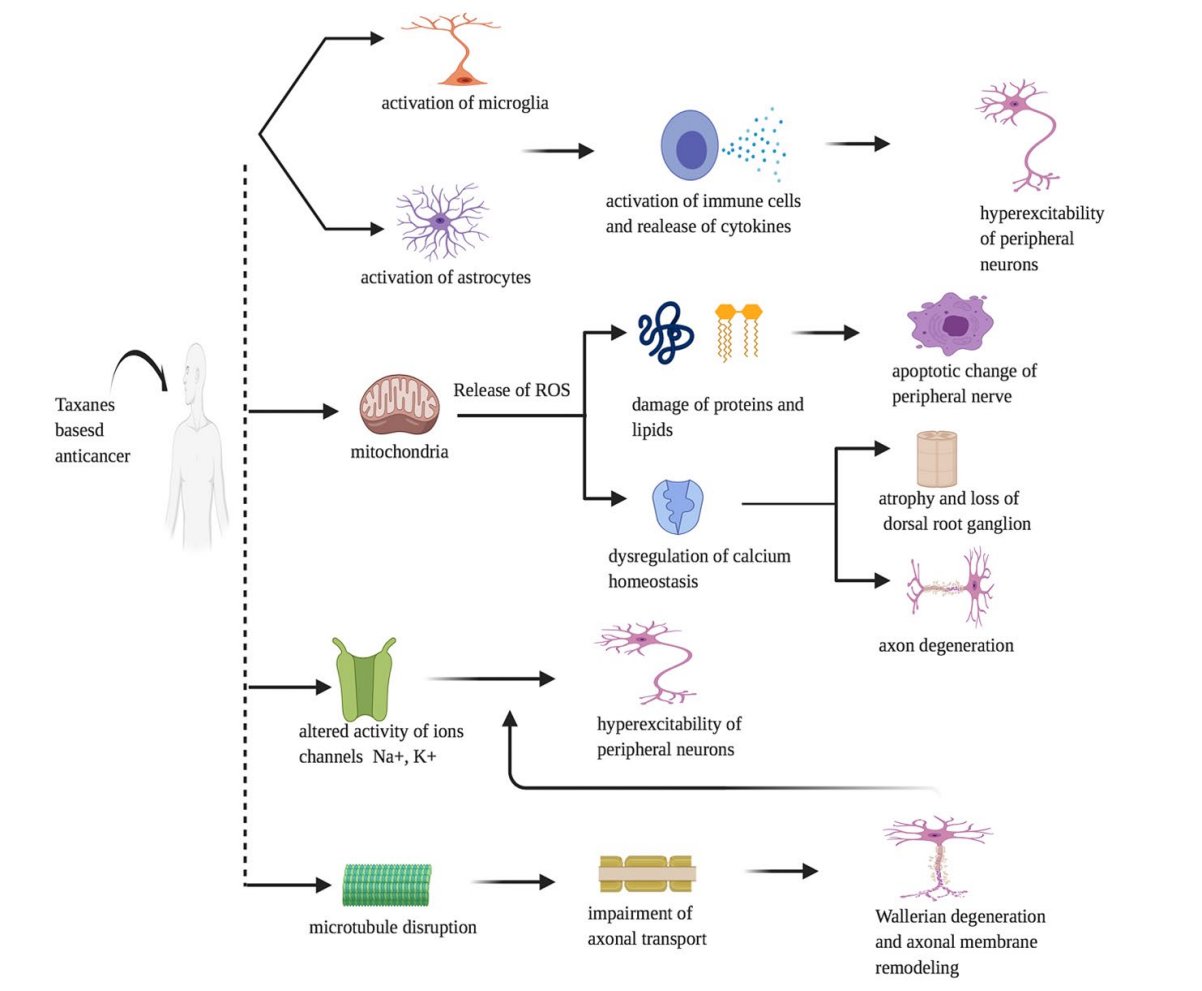
Pathophysiology of Taxanes-based antineoplastics-induced peripheral neuropathy. Effect of taxanes-based anticancer on microglia, astrocytes, mitochondria, ions channels and microtubule leading to peripheral neuropathy

The activation of microglia and astrocytes by taxanes also leads to the activation of immune cells and the release and elevation of pro-inflammatory cytokines (interleukins and chemokines), which results in the nociceptor sensitization and hyperexcitability of peripheral neurons. These processes lead to nociceptor sensitization and the development of neuroinflammation (Fig. 2), [18]. Epothilones, represented specifically by ixabepilone, an analog of epothilone B and sagopilone, are relatively new antineoplastic drugs with a similar mechanism to induce peripheral neuropathy as taxanes [19].

### Immunomodulatory drugs (thalidomide)

**Fig. 3:**
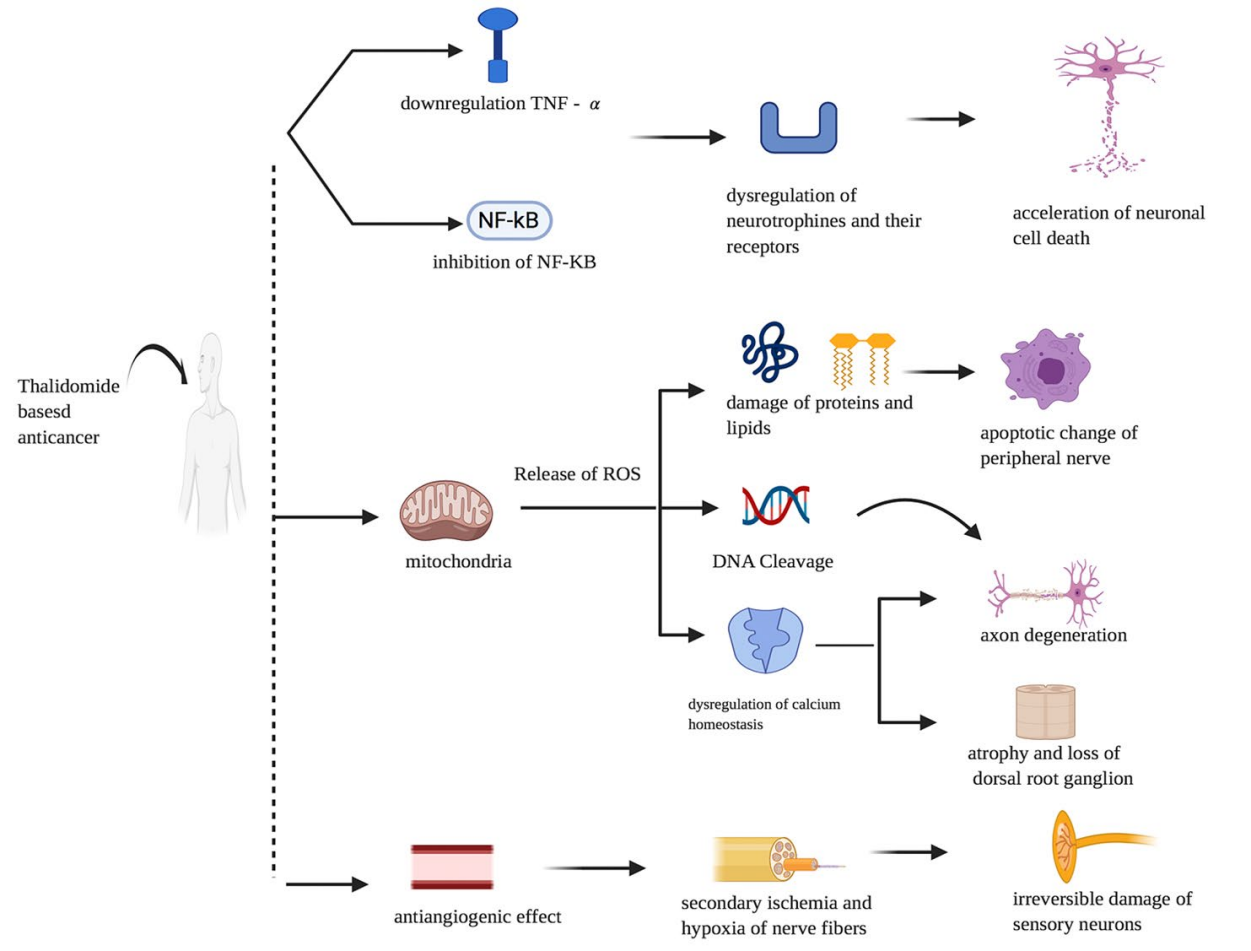
Pathophysiology of immunomodulatory-based antineoplastics-induced peripheral neuropathy. Effect of thalidomide-based anticancer on tumour necrosis factor, nuclear factor, mitochondria and angiogenesis leading to peripheral neuropathy

Thalidomide is a derivative of glutamic acid and an immunomodulatory medication approved for the treatment of multiple myeloma by the US Food and Drug Administration [20]. The mechanism of action of immunomodulatory drugs against cancer is little known. Thalidomide drugs blocks the production of tumor necrosis factor-alpha (TNF-α), and inhibits of activation of NF-kb (nuclear factor kappaB) followed by acceleration of neuronal cell death [21]. In addition, the thalidomide-induced antiangiogenic action induces secondary ischemia and nerve fibre hypoxia and, consequently, permanent sensory neuron harm [22].The activation of the thalidomide dihydroxide metabolite induces substantial release and activation of ROS and stimulates DNA cleavage (Fig. 3), but more preclinical and clinical studies are needed to validate the existence of such a function in peripheral neuropathy caused by thalidomide [23].

## Current treatments for chemotherapy-induced neuropathy

The majority of drug-induced peripheral neuropathies (DIPN) cause damage to the dorsal root ganglia and account for just 4% of all neuropathies, but DIPN can occur in 60% of patients undergoing chemotherapy [24, 25]. At present, the expert opinion of the American Society for Clinical Oncology’s 2014 Practice Guide has suggested that no agents can be approved for the prevention of CIPN and offers only modest support for treatment with a serotonin-norepinephrine reuptake inhibitor, duloxetine [11]. Unfortunately, duloxetine is associated with adverse effects (nausea, dizziness, and insomnia), and the prescription of the medication must be carefully controlled by a doctor [13]. As an alternative to duloxetine, tropical agents are sometimes recommended but have only symptomatic relief. Ketamine, baclofen, and amitriptyline topical application improved tingling sensory subscales but did not improve numbness, thermal pain, or functional abilities [26]. Due to a related overlapping process of mitotoxicity as part of pathophysiology, it is probable that a single pharmacological therapy may be possible for all CIPNs [27]. The existing medications approved for CIPN are only marginally successful in the management of symptoms of neuropathy. In fact, after the discontinuation of the medications, symptoms reappear. It has witnessed that presently available medications for the disease provide only short-term symptomatic relief and have significant adverse side effects. Unfortunately, there is no standard treatment procedure regarding CIPN prevention, mitigation, and management.

## Melittin as a potential drug for the chemotherapeutic-induced peripheral neuropathy

Honey bee venom (HBV) is a colourless, acidic liquid (pH 4.5–5.5) and the most valuable product of the honeybee. Its potential therapeutic value has been attributed to many diseases, particularly in rheumatic and arthritic conditions. HBV has at least 28 different active compounds with distinct health benefits. Several studies identified these compound’s biological properties and the health benefits of each ingredient. However, melittin is the main vital pharmacological component of HBV accounting for 40–50% dry weight of the venom [28]. It is a peptide with 26 amino acid residues and because of its amphiphilic nature, it is water-soluble [29]. If the allergenic components and histamine are removed, it is a safer product than whole bee venom [30, 31].

**Fig. 4:**
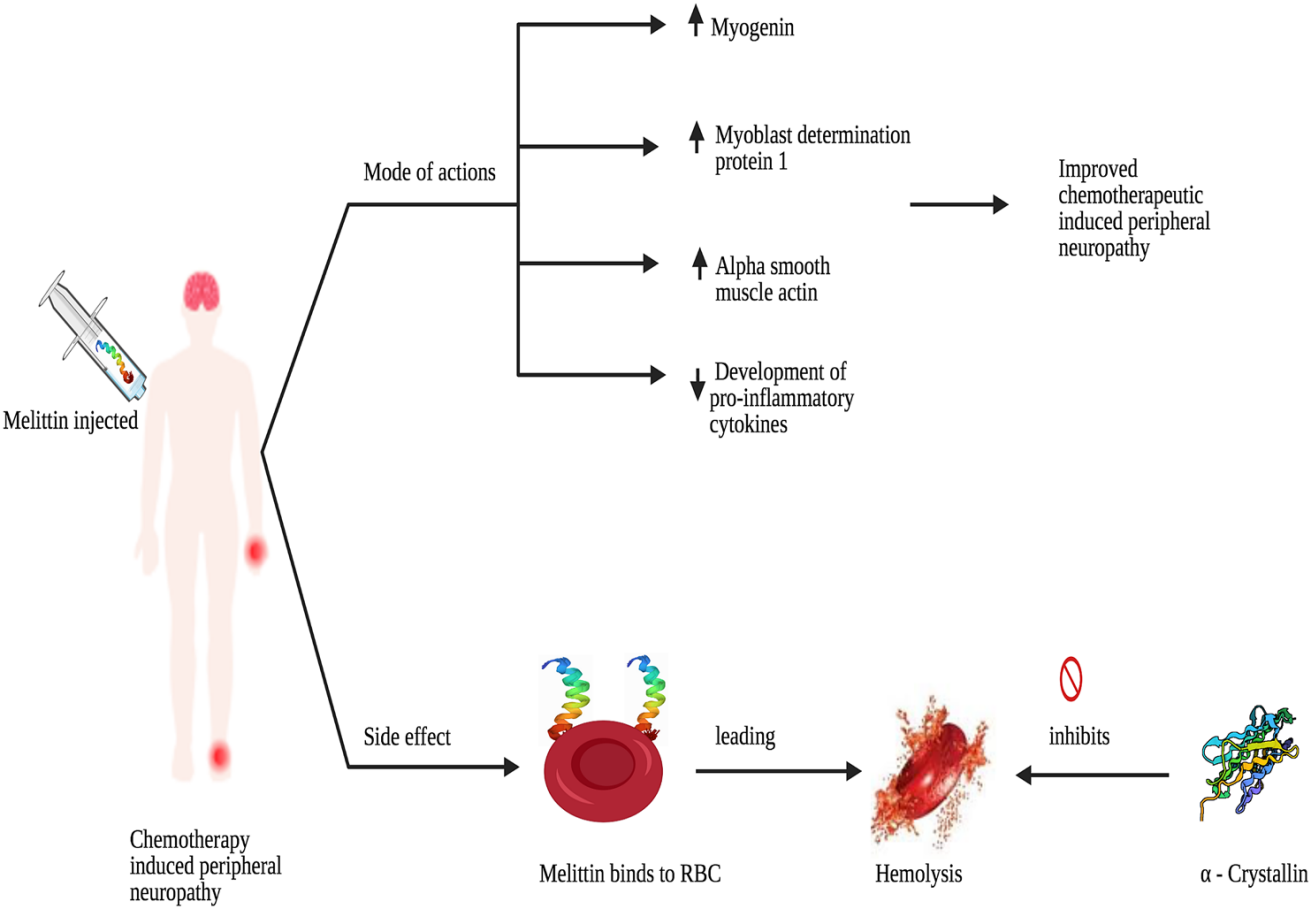
Possible mode of action of melittin on chemotherapy-induced peripheral neuropathy. Melittin elevates the expression of muscle regeneration factors like myogenin, myoblast determination protein 1 and alpha smooth muscle actin. It suppresses the development of pro-inflammatory cytokines and improves the histological changes of damaged tissue. However, hemolysis is the major concern with using melittin in chemotherapy-induced peripheral neuropathy patients. α-Crystallin may be used to inhibit the melittin-induced hemolysis

Melittin has many therapeutic values such as anti-viral, anti-bacterial, anti-fungal, anti-parasitic, anti-cancer and neuroprotective effect [32–35]. It strengthens muscle control, suppresses the development of pro-inflammatory cytokines, increases the expression of muscle regeneration factors (MyoD, myogenin, and α-SMA) and improved histological changes of damaged tissue in animal model of muscle contusion (Fig. 4) [36, 37]. In the studies conducted using different animal models of pain, HBV acupuncture has been reported to have a potent analgesic effect [34, 36, 38] and two studies have suggested that bee venom acupuncture treatment can help to reduce peripheral neuropathy caused by chemotherapy. Although there is no clinical trial evaluating the impact of melittin on humans, Park et al. examined the efficacy and protection of the sweet bee venom pharmacoacupuncture on five patients with peripheral neuropathy (CIPN) caused by chemotherapy [39, 40]. The graphic comparison scale of patients and the CIPN classification of the world health organization (WHO) as the primary outcomes shown that the treatment is effective without causing significant adverse effects such as allergic reaction. Since sweet bee venom therapy uses melittin, the discovery indicates that melittin can be used safely for future patients. When administered 20 min before treatment, prazosin (α1-adrenergic receptor antagonist, 30 μg, i.t.) or idazoxan (α2-adrenergic receptor antagonist, 50 μg, i.t.) blocked melittin analgesia on mechanical and cold allodynia. This suggested involvement in the melittin analgesic effects of both the spinal α1- and 2 adrenergic receptors [35]. Bee venom acupuncture combined with morphine has prolonged analgesic effects, in comparison to BVA or morphine alone. Similarly, it has shown that BVA could enhance the analgesic effect of clonidine injection in the persistent constriction-caused neuropathic pain model [41]. Whole honeybee venom attributes to allergic reaction whereas melittin is free from such action.

Because of its intense surface action on lipid membranes accompanied by the release of inflammatory mediators and the activation of primary nociceptor cells [34], melittin has thus far been considered a pain-producing agent. Subcutaneous injection of melittin on the back surface of rat’s hind paw actually produced spontaneous paw flinching reflex and an increase in the incidence of spontaneous spinal wide dynamic range (WDR) neuron discharges on the hind paw’s cutaneous receptive area. In contrast, subcutaneous melittin administration on the ST36 acupuncture point did not produce unwanted unpredictable behaviour. Injecting melittin at ST36 will contribute to more analgesic effect than systemic administration and the adverse effect associated with the melittin produced by injecting it through systemic injection such as intraperitoneal or intravenous can be avoided. Moreover, it did not generate spontaneous discharge of wide dynamic neuron that was stated to respond to the sensitive hind paw. Analgesic impact of acupuncture or electroacupuncture on ST36 was induced by triggering the descending pain inhibitory system [35], melittin injection on ST36 may also have triggered the descending noradrenergic inhibitory system. Melittin’s cytotoxicity depends on its dosage, considering its sensitivity to the erythrocyte membrane, low-dose intradermal, or subcutaneous injection of melittin may also be a safe method for haemolysis prevention [42].

## Limitation of melittin therapy

Despite having a strong effective report against chemotherapeutic-induced neuropathy, melittin was ruled out from clinical applicability since it induces haemolysis which might lead to rhabdomyolysis and acute renal failure resulting in a broad range of clinical signs starting from local oedemic swelling of skin to a life-threatening systemic anaphylactic shock [43]. These toxic features of melittin are indeed assumed as restricting factors for its use in cancer therapy [44]. Other reasons such as toxicity, lack of precision, degradation, systemic inefficiency, and limited bioavailability are also concerns for its therapeutic application [44–46]. To overcome these problems, various approaches including nanotechnology, gene therapy, and immunoconjugate were tried on melittin [30, 38]. However, the problem still persists and continues to be challenging [47]. Novel approaches are indeed necessary to tackle side effects associated with melittin treatments. In our study with melittin, we found that melittin-induced haemolysis in human RBCs can be inhibited by α-crystallin (human eye lens proteins). This study gives hope to use an appropriate amount of combination of melittin with α-crystallin to use it for therapeutic application without the problem of haemolysis.

## Novel approach to tackle melittin-induced haemolysis

Haemolysis is one of the major side effects of melittin which limits its usage in treating various diseases including chemotherapeutic-induced neuropathy. It was found that α-Crystallin (eye lens protein) can subdue the melittin-induced haemolysis in human RBC which indicates that melittin in combination with α-Crystallin can be useful in treating chemotherapeutic-induced neuropathy.

Crystallins are water-soluble chaperone proteins found in the lenses of all vertebrate eyes at high levels [48]. There are three classes of crystallins found in the human eye, namely α, β, and γ, which make up to more than 90% of the lens’ proteins [49]. α-Crystallin accounts for about 35% of all proteins of the vertebrate lens and is known to have chaperone-like properties and is a representative member of the small heat shock proteins (sHsp) [50–52]. It is understood that α-crystallin exhibits many physiological roles, i.e. preventing denatured protein precipitation and enhancing cellular tolerance to stress that are essential for maintenance of lens transparency and the prevention of cataract. Moreover, subunits of α-crystallin can inhibit cellular aggregation and protein inactivation in different stress states. It was discovered that melittin interacts with α crystallin with amino acid residues 13–21 and 71–88 of αA-crystallin and residues 70–78 of αB-crystallin [52].

### Prevention of haemolysis caused by melittin by α- crystallin

Heparinized human blood (expired for human transfusion) was collected from the blood bank of Kasturba Medical College (KMC), Manipal, Karnataka. The blood was centrifuged (3500 rpm, 10 min) and washed with PBS (×3). The Supernatant was disposed, and 2% RBC suspension was prepared with PBS. Melittin was diluted in PBS to get 8 μg/ml. Seven μg/ml of α-crystallin was used. Triton X-100 (1%) and phosphate buffer saline were used as positive and negative controls, respectively. The haemolytic activity was tested in a 96-well microtiter plate. Each well of the plate was added with 100 μl of 2% RBCs and treated with melittin alone and the combination of melittin and α-crystallin in 1:1 molar concentration. The plate was incubated (37 °C, 2 h) and centrifuged (3000 rpm, 10 min) at room temperature. Fifty μl of the supernatant was moved from each well into a fresh 96-well microtiter plate and at 540 nm optical density was measured using a microplate reader.

The percent of haemolysis was calculated as follows:

$${\rm{Haemolysis\, percentage}} = \frac{{\rm{OD\, of\, sample }} - {\rm{ OD\, of\, Neg}}.\, {\rm{control}}}{{\rm{OD\, Pos}}.\,{\rm{control}}-{\rm{OD\, Neg}}.\, {\rm{control}}} \times 10$$

**Fig. 5:**
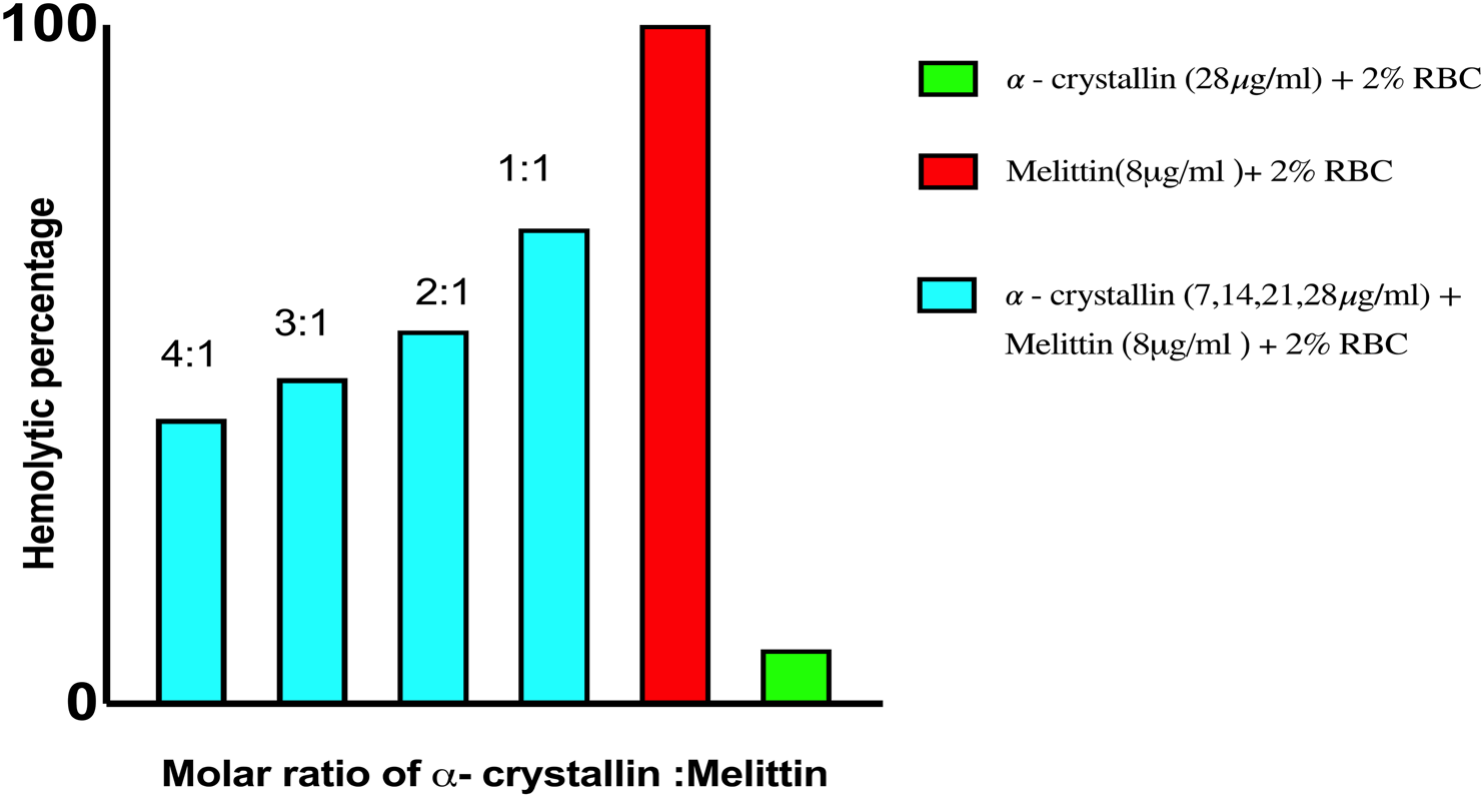
Represents the haemolytic percentage of Melittin and α-crystallin with RBC. α-Crystallin significantly decreases melittin-induced haemolysis

In accordance with the above study, we can clearly state that α-crystallin reduces melittin-induced haemolysis (Fig. 5). This data can be useful not only in the treatment of chemotherapeutic-induced neuropathy using melittin but also in the diseases where melittin has good therapeutic value. However, the therapeutic activity of melittin in presence of α-crystallin needs to be studied. Later appropriate combinations of α-crystallin and melittin may be used to restrain the therapeutic activity of melittin, at the same time the ill effects of it can be prevented.

## Conclusion

Whole honeybee venom and melittin are effective in treating few diseases. Particularly, in case of chemotherapy-induced neuropathy, melittin ameliorated the condition by improving mechanical hyperalgesia, cold allodynia and spinal wide dynamic range. Despite that, their usage has often been limited due to its side effects, mainly haemolysis. Since it is demonstrated that α-crystallin can inhibit melittin-induced haemolysis, it is plausible that this combination if used in appropriate ratios in the treatment of chemotherapeutic-induced neuropathy may have therapeutic benefit. Therefore, there is a need to further investigate the proposed combinational therapy with respect to its efficacy and safety.

## Data Availability

Not applicable.
